# Platform-specific comparison of IMRT and VMAT angular resolution for Ethos-based online adaptive radiotherapy in cervical cancer

**DOI:** 10.3389/fonc.2026.1888330

**Published:** 2026-08-05

**Authors:** Xiangyue Kong, Bin Zhang, Gongsen Zhang, Haonan Xiao

**Affiliations:** 1 Department of Radiation Oncology Physics and Technology, Shandong Cancer Hospital and Institute, Shandong First Medical University and Shandong Academy of Medical Sciences, Jinan, Shandong, China; 2 Artificial Intelligence Laboratory, Shandong Cancer Hospital and Institute, Shandong First Medical University and Shandong Academy of Medical Sciences, Jinan, Shandong, China

**Keywords:** angular resolution, cervical cancer, ethos, IMRT, online adaptive radiotherapy, VMAT

## Abstract

**Background:**

The Ethos™ platform enables online adaptive radiotherapy (oART) to mitigate inter-fractional anatomical variations. However, the lengthy “scan-to-beam” workflow introduces the risk of intra-fractional motion. This study aimed to investigate the dosimetric and efficiency differences between IMRT and VMAT with varying angular resolutions, providing data to guide the selection of planning strategies in Ethos-based oART.

**Materials and methods:**

Fifteen patients with cervical cancer treated on the Ethos platform were retrospectively analyzed. For each patient, five plans were generated: a standard 9-field IMRT plan and four VMAT plans optimized with angular resolutions of 2°, 3°, 4°, and 5° (VMAT_2_-VMAT_5_). Dosimetric metrics for Planning Target Volume (PTV) and Organs at Risk (OARs), along with plan generation efficiency (optimization and calculation time), delivery efficiency (beam-on and total delivery time), and deliverability (gamma analysis), were systematically evaluated.

**Results:**

All plans met clinical requirements. IMRT demonstrated superior dose homogeneity (HI: 0.07 ± 0.01) compared to all VMAT groups (0.09-0.10, *P< 0.001*). For OARs, IMRT provided better sparing of the rectum, bladder, and bone marrow, and reduced the high-dose bowel metric D2cc; however, bowel V_30_ was lower in the VMAT groups, indicating metric-dependent bowel sparing. In terms of efficiency, IMRT required substantially shorter optimization time (122.8 ± 14.6 s) than VMAT (705.9-742.9 s, *P< 0.001*). Conversely, VMAT reduced the total delivery time by approximately 50% compared to IMRT (158.1 s vs. 305.3 s, *P< 0.001*). Among VMAT plans, finer resolutions (2°-3°) offered slight dosimetric advantages over coarser resolutions (4°-5°) but required longer calculation times. Gamma passing rates were >98% (3%/3mm) for all techniques, with IMRT showing marginally higher accuracy.

**Conclusion:**

On the Ethos platform, the IMRT technique offers a favorable balance for cervical cancer oART, combining superior target homogeneity, improved rectum, bladder, and bone marrow sparing, lower bowel D_2cc_, and rapid plan generation that minimizes the critical scan-to-beam interval. However, bowel V_30_ was lower with VMAT, suggesting that bowel sparing depends on the selected dose-volume metric.

## 1 Introduction

Cervical cancer remains a leading gynecological malignancy, with definitive radiotherapy being a cornerstone of curative management (1). Modern delivery techniques, such as Intensity-Modulated Radiotherapy (IMRT) and Volumetric Modulated Arc Therapy (VMAT), have become the standard of care. By creating steep dose gradients, these techniques allow for the escalation of dose to the target volume while maximizing the sparing of adjacent Organs at Risk (OARs), specifically the bladder, rectum, and bowel (2–4).

However, the delivery of high-precision radiotherapy to the pelvis is complicated by significant anatomical variability. The highly deformable nature of the uterus and cervix, coupled with random variations in bladder filling and rectal distension, leads to substantial inter-fractional motion (5). Consequently, a static treatment plan generated from a simulation CT often fails to represent the patient’s daily anatomy (6). To address this challenge, the Ethos™ platform (Varian Medical Systems, Palo Alto, CA) was developed, enabling Online Adaptive Radiotherapy (oART). Leveraging on-board AI-driven segmentation, this commercially available system allows for the re-optimization of a plan adapted to the daily anatomy within a standard treatment slot (7, 8).

While online adaptation effectively mitigates inter-fractional errors, the workflow introduces a secondary challenge: intra-fractional motion. As the patient remains immobilized on the treatment couch during contouring and planning, physiological processes (e.g., bladder filling) continue to alter the internal anatomy (9). Therefore, minimizing the total time from scanning to beam completion is a dosimetric necessity. This efficiency depends on two factors: generation time and delivery time. Currently, the widely adopted clinical standard for Ethos-based cervical cancer treatment - and the protocol followed at our institution - is the 9-field equidistant IMRT technique. While this approach is known to ensure robust plan quality, VMAT strategies theoretically offer potential advantages in delivery efficiency, their optimal implementation on the Ethos platform remains underinvestigated.

For VMAT planning on the Ethos Intelligent Optimization Engine (IOE), a critical parameter governing both calculation and delivery efficiency is the angular resolution. This parameter dictates the interval at which dose is calculated and MLC leaves are positioned. A finer resolution (2° or 3°) increases the number of control points, which may improve dose conformity but significantly prolongs optimization time and potentially increases plan complexity (total monitor units), leading to slower delivery. Conversely, a coarser resolution (4° or 5°) accelerates calculation, the trade-off in dosimetric quality - especially when compared to the established IMRT standard-is not well understood.

Although the evaluated IMRT and VMAT configurations are available within the commercial Ethos planning environment, quantitative evidence regarding their relative dosimetric and temporal performance in cervical cancer remains limited, particularly when different VMAT angular resolutions are evaluated under otherwise identical planning conditions. Therefore, the purpose of this study was not to develop a new planning technique, but to provide a controlled, platform-specific comparison of the manufacturer-defined 9-field IMRT configuration and VMAT plans generated with angular resolutions of 2°, 3°, 4°, and 5°. Using the same patient anatomy, isocenter setting, clinical goals template, dose-calculation parameters, and automated optimization workflow, we evaluated target and OAR dosimetry, optimization and dose-calculation time, treatment-delivery efficiency, monitor units, overall workflow time, and secondary dose verification. This design was intended to quantify the practical trade-offs between plan quality and workflow efficiency within the current Ethos software environment and to provide a reproducible technical benchmark for institutions considering these planning options in cervical cancer oART.

## 2 Materials and methods

### 2.1 Patient selection and data acquisition

This retrospective study was approved by the Institutional Review Board of Shandong Cancer Prevention and Treatment Research Institute (Ethical Approval No: SDTHEC202410027). Data were collected from 15 patients with histopathologically confirmed cervical carcinoma treated at our institution between January 2024 and October 2025. Eligible patients met the following inclusion criteria: (1) treatment with definitive radiotherapy using the Ethos™ platform; (2) a prescribed dose of 50.4 Gy in 28 fractions (1.8 Gy/fraction); and (3) complete CT simulation datasets available for retrospective replanning. Patients with previous pelvic irradiation or hip prostheses causing severe metal artifacts were excluded. The median age of the cohort was 59 years (range, 33–70 years). The clinical characteristics of the patients are summarized in **Table 1**.

**Table 1:** Characteristics of the patients.

Characteristics	Patients (n = 15)
Median age (range) - yr	59 (33-70)
Stage I	6 (40.0%)
IB2	3 (20.0%)
IB3	3 (20.0%)
Stage II	3 (20.0%)
IIA	2 (13.3%)
IIB	1 (6.7%)
Stage III	6 (40.0%)
IIIC1	6 (40.0%)
Median PTV volume (range) - cm^3^	1275 (1017.5 - 1626.1)

PTV, planning target volume.

### 2.2 CT simulation and delineation

All patients underwent computed tomography (CT) simulation in the supine position. Immobilization was achieved using a vacuum-formed cushion to ensure reproducibility. CT scans were acquired using a SOMATOM Confidence CT simulator (Siemens Healthineers, Forchheim, Germany) with a slice thickness of 3 mm. Images were transferred to the Varian Eclipse™ treatment planning system (TPS) (version 16.1, Varian Medical Systems, Palo Alto, CA, USA) for contouring. The clinical target volume (CTV) and organs at risk (OARs) were delineated by experienced radiation oncologists following the RTOG consensus guidelines (10, 11). The CTV included the gross tumor volume, cervix, uterus, parametria, and regional lymph nodes. A uniform margin of 5–7 mm was added to the CTV to generate the planning target volume (PTV). OARs contoured included the bladder, rectum, bowel, femoral heads, marrow.

### 2.3 Treatment planning strategies

CT scan images and structure contours were exported from the Eclipse TPS to the Ethos TPS (version 02.01.00; Varian Medical Systems, Palo Alto, CA, USA) for retrospective planning. All VMAT plans were created retrospectively for study purposes. Patients were not treated with these plans. The Ethos platform is based on an O-ring gantry linear accelerator design and is equipped with a 6-MV flattening-filter-free (FFF) beam, a dual-layer stacked multi-leaf collimator (MLC), a maximum dose rate of 800 MU/min, and a maximum treatment field size of 28 × 28 cm² at the isocenter.

For each patient, five treatment plans were generated using the commercially available planning functions of the Ethos Intelligent Optimization Engine (IOE). The IOE uses an automated multi-criteria optimization framework driven by prioritized clinical goals. The investigated planning techniques were not newly developed for this study. Rather, they represented manufacturer-defined or commercially available planning options within the Ethos environment that were applied under a standardized study protocol. To ensure comparability, all plans used the same patient anatomy, isocenter location at the geometric center of the PTV, clinical goals template, and dose-calculation grid of 2.5 mm, without manual intervention during optimization.

Planning was driven by an identical prioritized clinical goals template for all five planning strategies. The template included PTV coverage and high-dose control goals, together with dose–volume constraints for the bowel, rectum, bladder, marrow, and bilateral femoral heads. The same goals and priority order were applied to the IMRT and VMAT_2_–VMAT_5_ plans without manual modification during optimization. A complete description of the clinical goals and priority levels is provided in **Supplementary Table 1**.

The fixed-field IMRT plans used the manufacturer-defined default Ethos configuration of nine equidistant coplanar fields at gantry angles of 20°, 60°, 100°, 140°, 180°, 220°, 260°, 300°, and 340°, with a collimator angle of 10°for all fields. For the VMAT comparison, the commercially available Ethos VMAT planning option was applied using an identical dual-arc full-rotation trajectory with collimator angles of 15° and 345°. The study-specific decision was to systematically evaluate four selectable angular resolutions—2°, 3°, 4°, and 5°—while keeping all other planning parameters unchanged. These plans were designated VMAT_2_, VMAT_3_, VMAT_4_, and VMAT_5_, respectively. Thus, the comparison evaluated the effects of delivery technique and angular resolution rather than newly developed beam configurations or optimization methods.

### 2.4 Evaluation of metrics

#### 2.4.1 Dosimetric indices

Dosimetric metrics were extracted from the cumulative dose-volume histograms (DVH). Target coverage and dose distribution were evaluated using the following metrics: D_98%_: near-minimum dose. D_2%_: near-maximum dose. D_50%_: median dose. D_mean_: mean dose. To quantify plan quality, the following indices were calculated: the conformity index (CI), calculated using the Paddick formula to assess how well the prescription isodose conforms to the target shape (**Equation 1**), and the homogeneity index (HI), calculated to evaluate dose uniformity within the target (**Equation 2**) (12, 13). The calculation formula is as follows:

(1) $$\mathrm{HI}=\frac{D_{2\%}-D_{98\%}}{D_{50\%}}$$

where D_2%_, D_98%_, D_50%_ were the doses received by 2%, 50% and 98% of the PTV, respectively.

(2) $$CI=\frac{{\left({TV}_{PV}\right)}^{2}}{TV\cdot V_{PV}}$$

where TV denoted the volume of the PTV, TV_PV_ represents the target volume covered by the prescription isodose, and V_PV_ denoted the total volume covered by the prescription isodose.

Dosimetric parameters for OARs were evaluated, including the mean dose (D_mean_), near-maximum dose (D_2cc_), and the percentage of volume receiving > x Gy (V_x_). For Bladder and Rectum: V_40Gy_, D_mean_. Bowel: V_30Gy_, D_2cc_. Marrow: V_30Gy_, D_90%_. Femoral Heads: V_40Gy_, D_mean_.

#### 2.4.2 Efficiency assessment

To comprehensively evaluate clinical workflow efficiency, four distinct time metrics were recorded for each plan. Plan generation efficiency was assessed using: (1) automatic optimization time, defined as the computational time required by the IOE to perform the optimization process; and (2) dose calculation time, the duration required for the final dose computation post-optimization. Delivery efficiency was evaluated using: (3) beam-on time, representing the actual duration of radiation output; and (4) total delivery time, the estimated total treatment execution time, accounting for gantry rotation and MLC movement. In addition, the study-defined overall workflow time was calculated for each individual plan as the sum of optimization time, dose-calculation time, and delivery time. Total monitor units (MUs) were recorded to assess overall machine output and plan complexity. In addition, Mean MU per field/arc (Average MU) was calculated as total MU divided by the number of IMRT fields or VMAT arcs. Specifically, total MU was divided by 9 for IMRT plans and by 2 for VMAT plans. This metric describes the average machine output assigned to each treatment field or arc and should not be interpreted as MU per control point.

#### 2.4.3 Plan deliverability verification

Plan deliverability and accuracy were verified using the Mobius3D secondary dose calculation software (Varian Medical Systems, Palo Alto, CA, USA). Gamma analysis was performed using three evaluation criteria: 2%/2 mm, 3%/3 mm, and 5%/5 mm. The 3%/3 mm criterion was used as the conventional clinical benchmark, whereas the more stringent 2%/2 mm criterion was included to provide greater sensitivity for detecting differences in secondary dose-calculation agreement among the planning strategies. The 5%/5 mm criterion was retained as a permissive reference for identifying gross discrepancies rather than as the principal comparative endpoint. Gamma passing rates were recorded for all plans under each criterion.

### 2.5 Statistical analysis

All data were input into and analyzed by using Statistical Product and Service Solutions (IBM SPSS 26.0; IBM Corp., Armonk, NY). Quantitative data were expressed as the mean ± standard deviation. For multi-group comparisons of dosimetric parameters and delivery efficiency, Repeated Measures ANOVA was applied for normally distributed data, while the Friedman test was used for non-normally distributed data. *Post-hoc* pairwise comparisons were conducted using the Bonferroni correction to control for Type I error, with a statistically significant level set at *P< 0.005* (calculated as *0.05/10* for ten pairwise combinations).

## 3 Results

**Figure 1:**
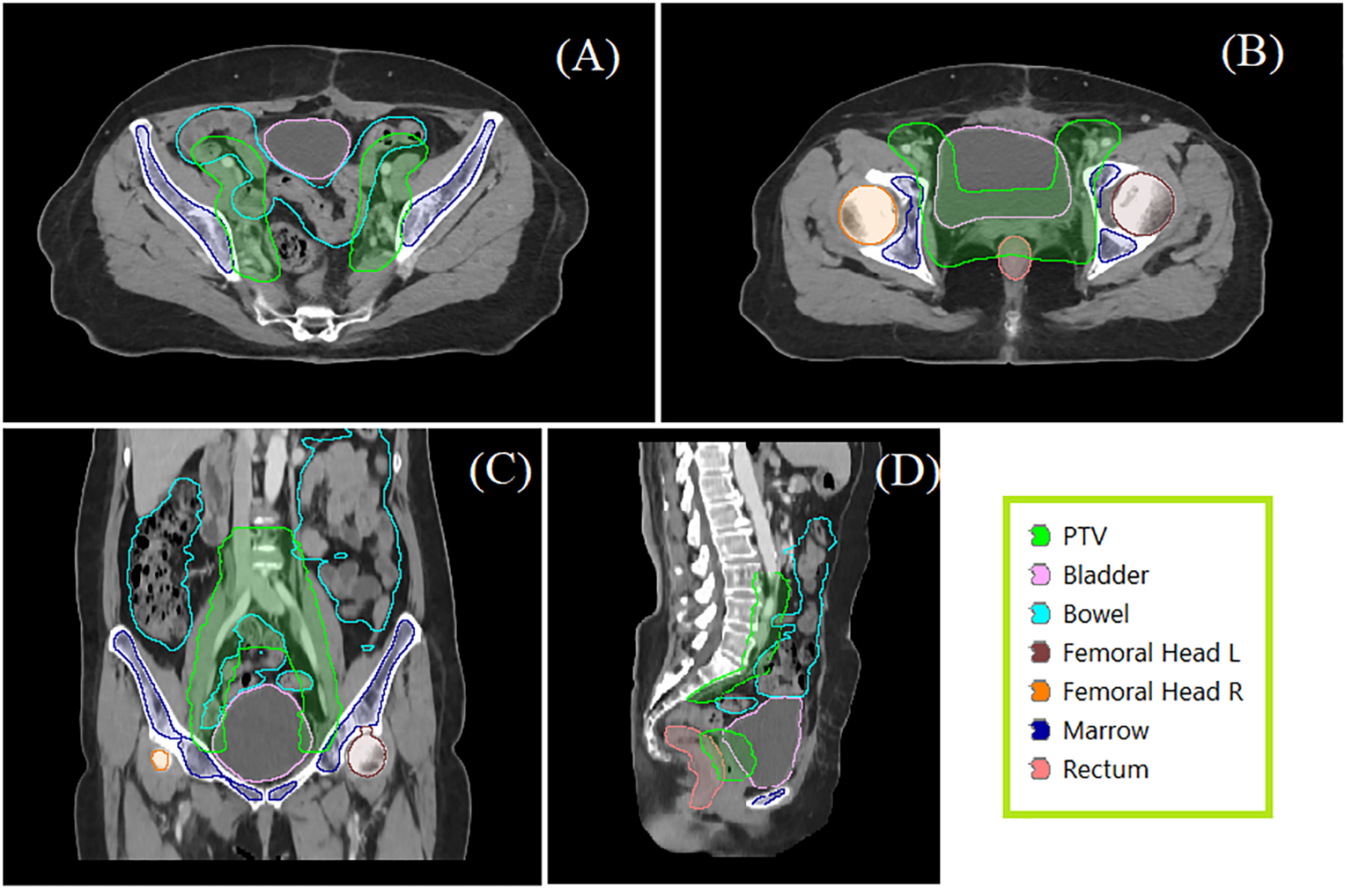
Planning CT images of a representative patient with target and organ-at-risk delineation. The planning target volume (PTV) and relevant organs at risk, including the bladder, bowel, rectum, bone marrow, and bilateral femoral heads, are shown in axial **(A, B)**, coronal **(C)**, and sagittal **(D)** views. A color-coded legend is provided for clarity.

**Figure 2:**
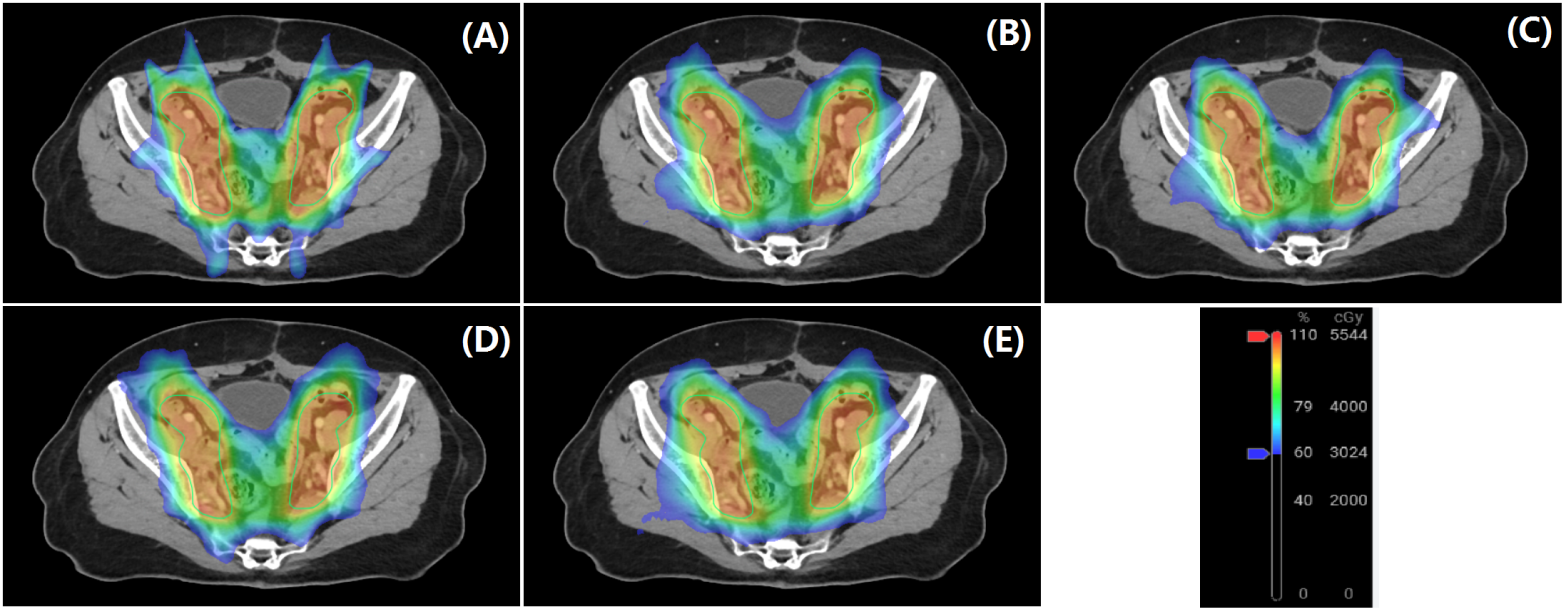
Comparison of axial dose distributions for a representative patient. **(A)** Standard IMRT plan. **(B–E)** VMAT plans optimized with angular resolutions of 2° (VMAT_2_), 3° (VMAT_3_), 4° (VMAT_4_), and 5° (VMAT_5_). The green contour indicates the Planning Target Volume (PTV).

**Figure 3:**
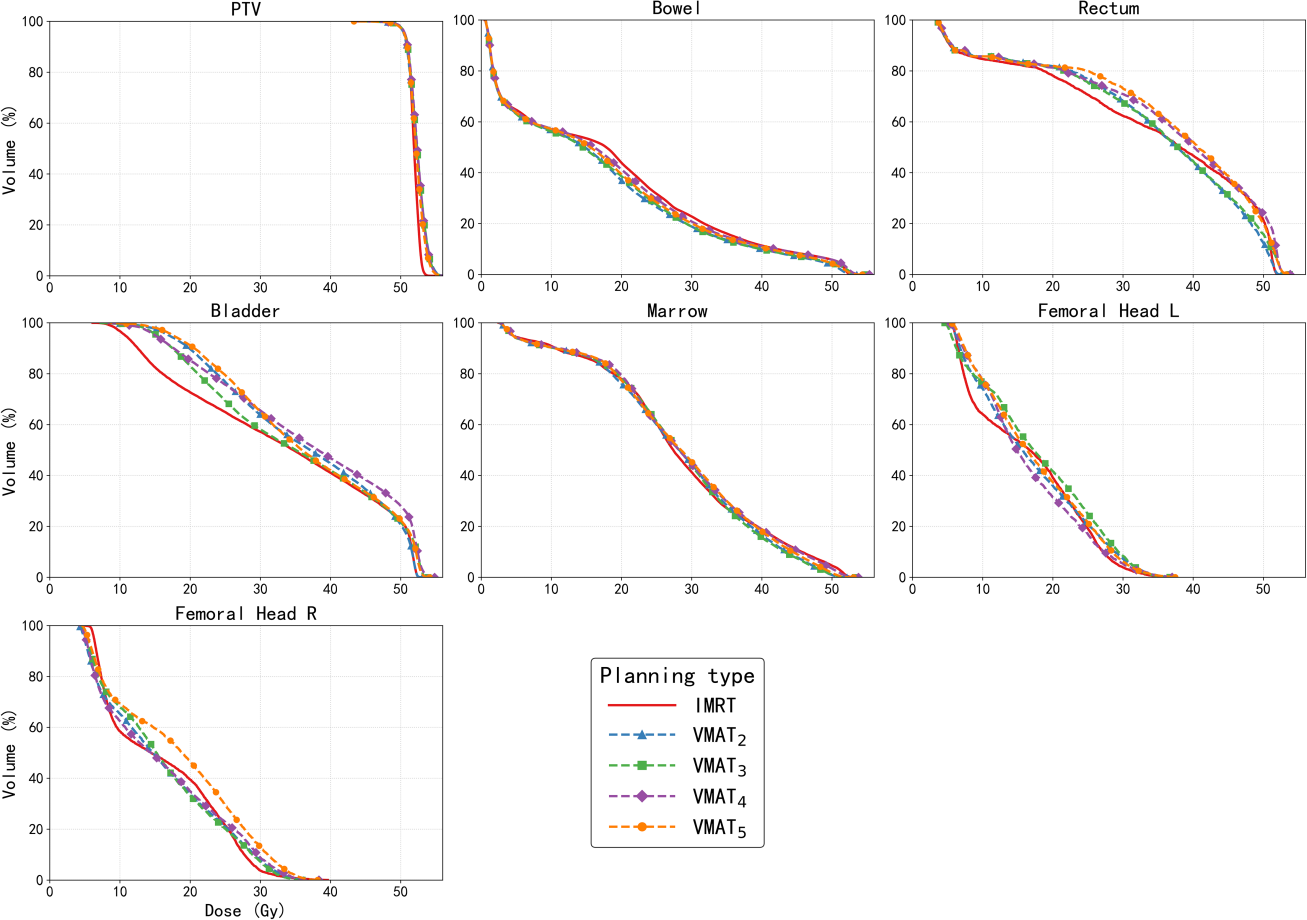
Cumulative Dose-Volume Histograms (DVH) for the PTV and OARs in a representative patient.

Clinically acceptable treatment plans were successfully generated for all patients using the standard IMRT technique and VMAT strategies with varying angular resolutions. All plans met the prescribed PTV coverage and OAR-sparing requirements. **Figure 1** shows the planning CT of a representative patient with the delineated PTV and relevant OARs in axial, coronal, and sagittal views, providing anatomical context for the subsequent dosimetric comparison. **Figure 2** presents the corresponding axial dose distributions for the five planning strategies, demonstrating comparable target conformity across techniques. The cumulative dose–volume histograms (DVHs) are shown in **Figure 3** and illustrate the differences in target dose distribution and OAR sparing between the IMRT and VMAT plans.

### 3.1 Target coverage

The dosimetric results for the PTV across the five planning groups are presented in **Table 2**, respectively. All planning groups achieved satisfactory target coverage, with no significant differences observed in V_100%_ (*P = 0.00*6), D_98%_ (*P = 0.632*), D_mean_ (*P = 0.013*), and the CI, (*P = 0.914*). However, the IMRT plans demonstrated a significantly lower Homogeneity Index (HI) (0.07 ± 0.01) compared to all VMAT plans (*P< 0.001*), indicating superior dose homogeneity. Additionally, D_2%_ in the IMRT group was significantly lower than in the VMAT_3_, VMAT_4_, and VMAT_5_ groups (*P< 0.001*). Among the VMAT groups, no statistically significant differences were observed in most PTV dosimetric parameters (*P > 0.005*), with the exception of D_2%_, where VMAT_2_ demonstrated a significantly lower dose compared to VMAT_3_ (*P< 0.005*).

**Table 2:** Dosimetric comparison of PTV coverage, conformity, and homogeneity between IMRT and VMAT plans with different angular resolutions.

Metrics	IMRT	VMAT_2_​	VMAT_3_​	VMAT_4_​	VMAT_5_​	*P*-value
V_100%_ (%)	96.66 ± 0.67^a^	95.82 ± 0.57^a^	96.07 ± 0.72^a^	96.27 ± 1.13^a^	95.99 ± 0.74^a^	0.006
D_98%_ (Gy)	49.81 ± 0.38^a^	49.71 ± 0.26^a^	49.70 ± 0.37^a^	49.84 ± 0.41^a^	49.72 ± 0.30^a^	0.632
D_2%_ (Gy)	53.57 ± 0.48^c^	53.95 ± 0.52^bc^	54.22 ± 0.49^a^	54.20 ± 0.47^ab^	54.13 ± 0.44^ab^	< 0.001
D_mean_ (Gy)	52.08 ± 0.33^a^	52.20 ± 0.38^a^	52.44 ± 0.41^a^	52.32 ± 0.34^a^	52.37 ± 0.43^a^	0.013
HI	0.07 ± 0.01^b^	0.09 ± 0.01^a^	0.10 ± 0.01^a^	0.09 ± 0.01^a^	0.09 ± 0.01^a^	< 0.001
CI	0.89 ± 0.02^a^	0.89 ± 0.03^a^	0.90 ± 0.02^a^	0.89 ± 0.01^a^	0.89 ± 0.03^a^	0.914

Data are presented as mean ± standard deviation. V_100%_ (%) is the percentage of PTV volume receiving 100% of the prescribed dose; D_98%_ and D_2%_ are the doses received by 98% and 2% of the PTV volume, respectively. HI, Homogeneity Index; CI, Conformity Index. *P*-values were determined by Repeated Measures ANOVA or the Friedman test. Within each row, means labeled with different superscript letters (^a,b,c^) indicate a statistically significant difference (*P< 0.005*, Bonferroni corrected).

Overall, all five planning strategies achieved comparable PTV coverage and conformity, as reflected by the absence of significant differences in V_100%_, D_98%_, D_mean_, and CI. The principal advantage of the default IMRT configuration was improved dose homogeneity and lower near-maximum target dose. Among the VMAT strategies, VMAT_2_ provided the most favorable high-dose control and was closest to IMRT, whereas VMAT_3_–VMAT_5_ showed slightly higher D_2%_ and HI values without a clear improvement in target coverage or conformity. Nevertheless, the absolute differences were modest, and all strategies satisfied the predefined clinical target requirements.

### 3.2 OAR sparing

The dosimetric comparisons for OARs among the five planning groups are detailed in **Table 3**. Significant statistical differences were observed in specific dose-volume metrics depending on the organ type.

**Table 3:** Comparison of dosimetric parameters for organs at risk (OARs) between IMRT and VMAT plans with different angular resolutions.

OARs	Metrics	IMRT	VMAT_2_	VMAT_3_	VMAT_4_	VMAT_5_	*P*-value
Bowel	V_30_​ (%)	22.47 ± 6.95^b^	20.50 ± 7.06^a^	20.14 ± 7.10^a^	20.42 ± 6.97^a^	20.52 ± 7.13^a^	< 0.001
	D_2cc_ (Gy)	52.33 ± 0.45^c^	53.24 ± 0.72^a^	53.50 ± 0.73^ab^	53.41 ± 0.68^ab^	53.66 ± 0.69^b^	< 0.001
Rectum	D_mean_​ (Gy)	38.10 ± 1.83^b^	39.44 ± 2.13^a^	39.41 ± 2.27^a^	39.48 ± 2.15^a^	39.80 ± 2.11^a^	< 0.001
	V_40_​ (%)	49.85 ± 3.09^c^	52.17 ± 3.82^a^	52.60 ± 4.23^a^	53.67 ± 2.98^ab^	54.91 ± 2.53^b^	< 0.001
	D_2cc_​ (Gy)	51.77 ± 0.44^a^	52.00 ± 0.43^ab^	52.13 ± 0.46^ab^	52.25 ± 0.42^ab^	52.32 ± 0.39^b^	0.001
Bladder	D_mean_​ (Gy)	36.73 ± 2.24^b^	38.06 ± 2.28^a^	37.88 ± 2.31^a^	38.86 ± 3.59^a^	38.24 ± 2.36^a^	< 0.001
	V_40_​ (%)	44.60 ± 6.27^b^	48.05 ± 7.19^a^	47.26 ± 7.58^a^	48.19 ± 6.99^a^	48.03 ± 7.73^a^	< 0.001
	D_2cc_​ (Gy)	52.20 ± 0.32^b^	52.95 ± 0.59^a^	53.04 ± 0.48^a^	53.24 ± 0.71^a^	53.28 ± 0.59^a^	< 0.001
Marrow	V_30_​ (%)	48.60 ± 4.99^b^	51.77 ± 5.56^a^	52.31 ± 5.32^a^	51.97 ± 5.61^a^	52.46 ± 5.16^a^	< 0.001
	D_90%_​ (Gy)	15.44 ± 2.22^a^	16.17 ± 2.06^ab^	16.30 ± 2.02^abc^	16.52 ± 1.90^c^	16.48 ± 2.16^bc^	0.006
Femur Left	D_mean_​ (Gy)	19.91 ± 1.44^a^	20.13 ± 1.25^a^	20.14 ± 1.39^a^	20.24 ± 1.50^a^	20.50 ± 1.37^a^	0.211
	V_40_​ (%)	1.05 ± 0.86^a^	1.26 ± 0.99^a^	1.40 ± 1.08^a^	1.47 ± 1.21^a^	1.56 ± 1.34^a^	0.105
Femur Right	D_mean_​ (Gy)	19.75 ± 2.05^a^	20.31 ± 2.00^ab^	20.30 ± 1.77^ab^	20.42 ± 1.99^ab^	20.83 ± 1.75^b^	0.003
	V_40_​ (%)	1.13 ± 0.97^a^	1.42 ± 0.91^ab^	1.48 ± 0.93^ab^	1.39 ± 1.03^ab^	1.93 ± 1.18^b^	0.023

Data are presented as mean ± standard deviation. D_mean_, mean dose; D_2cc_, the dose received by the most irradiated 2 cm³ volume; V_x_, the percentage of the organ volume receiving at least x Gy. *P*-values were determined by Repeated Measures ANOVA or the Friedman test. Within each row, means labeled with different superscript letters (^a,b,c^) indicate a statistically significant difference (*P< 0.005*, Bonferroni corrected).

For the bowel, the IMRT group exhibited a significantly higher V_30_ (22.47 ± 6.95%) compared to all VMAT planning groups (20.14% - 20.52%, *P< 0.001*). Conversely, in the high-dose region, the IMRT group demonstrated significantly lower values for D_2cc_ compared to the VMAT groups (*P< 0.001*). No significant differences were found among the VMAT groups for these metrics (*P > 0.005*). Regarding the rectum, the IMRT plan resulted in significantly lower D_mean_, V_40_, and D_2cc_ compared to the VMAT plans (*P ≤ 0.002*). Similarly, for the bladder, the IMRT group showed significantly lower values for D_mean_, V_40_, and D_2cc_ than all VMAT groups (*P< 0.001*). The dosimetric parameters for both the rectum and bladder were comparable across the four VMAT groups, with no statistically significant differences observed (*P > 0.005*). For marrow sparing, the IMRT group achieved a significantly lower V_30_ (48.60 ± 4.99%) compared to the VMAT groups (*P< 0.001*). The D_90%_ of the marrow was also lowest in the IMRT group (15.44 ± 2.22 Gy), showing a significant difference compared to VMAT_4_ (*P< 0.005*). For the femoral heads, no significant differences were observed in D_mean_ and V_40_ for the left femoral head across all five groups (*P ≥ 0.105*). For the femoral head right, the IMRT group achieved a significantly lower D_mean_ (19.75 ± 2.05 Gy) compared to the VMAT_5_ group (*P = 0.003*), while no significant differences were found among the VMAT_2_, VMAT_3_, and VMAT_4_ groups.

Furthermore, contrary to the overall trend, some OAR metrics showed subtle but significant variations among VMAT groups; for instance, VMAT_2_ demonstrated significantly lower D_2cc_ for the bowel and V_40_ for the rectum compared to VMAT_5_ (*P< 0.005*).

Although several OAR differences reached statistical significance, the absolute magnitudes were modest for some endpoints. These findings should therefore be interpreted as dosimetric planning differences rather than direct evidence of reduced clinical toxicity. Nevertheless, even modest differences in cumulative high-dose exposure to the rectum, bladder, and bowel may have potential clinical relevance, while the reduction in pelvic marrow dose may be particularly relevant for patients receiving concurrent chemotherapy. Prospective clinical outcome data are required to determine whether these dosimetric differences translate into meaningful toxicity reduction.

### 3.3 Planning and delivery efficiency

**Figure 4:**
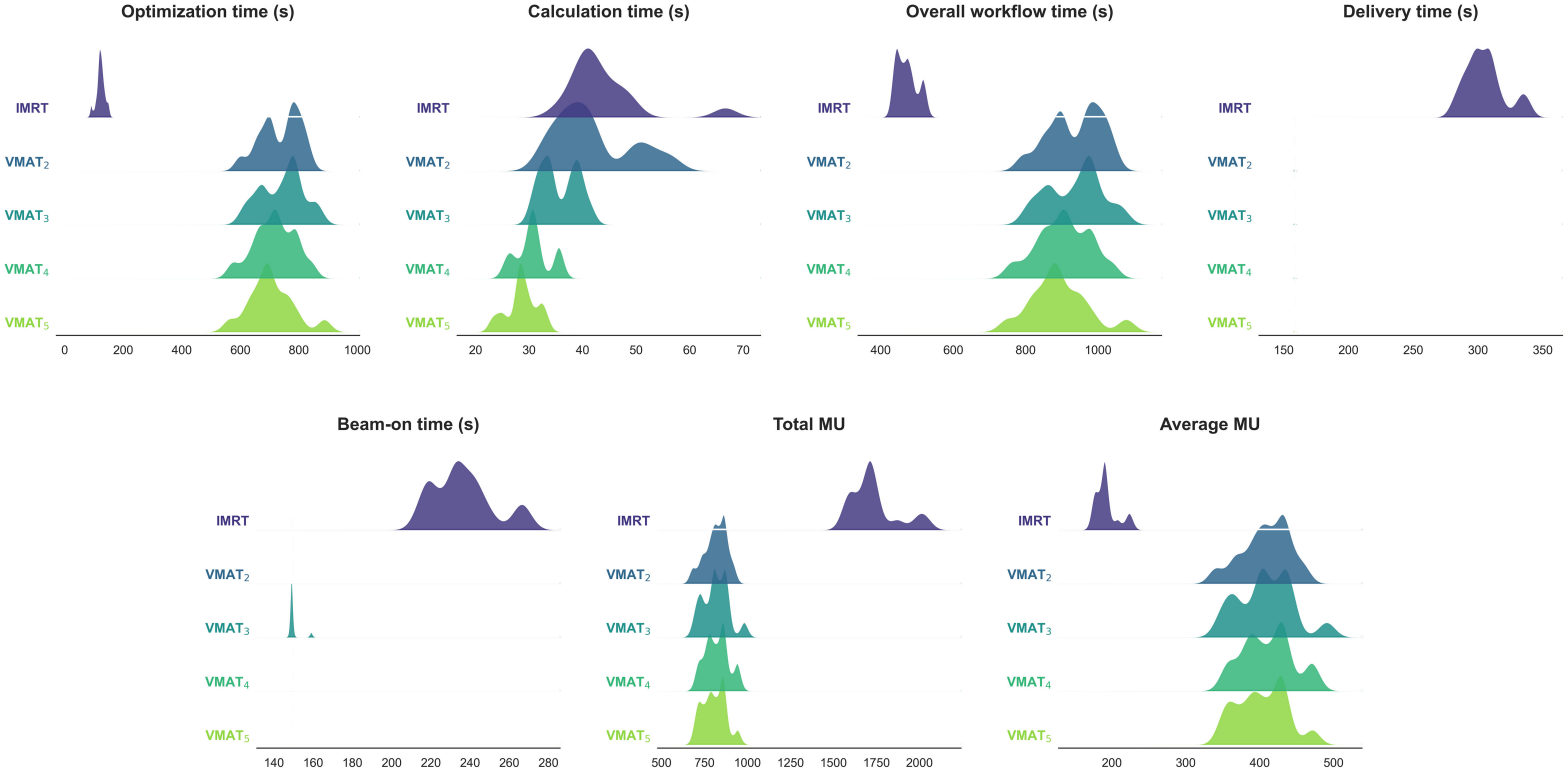
Ridgeline plots of planning efficiency and delivery parameters for IMRT and VMAT groups.

The comparison of planning efficiency and delivery parameters among IMRT and VMAT groups is summarized in **Table 4**, with their corresponding distribution patterns illustrated in the ridgeline plots (**Figure 4**).

**Table 4:** Comparison of planning efficiency and delivery parameters across all planning groups.

Metrics	IMRT	VMAT_2_	VMAT_3_	VMAT_4_	VMAT_5_	*P*-value
Optimization time (s)	122.8 ± 14.6^a^	736.9 ± 68.5^b^	742.9 ± 74.8^b^	716.7 ± 71.6^b^	705.9 ± 79.1^c^	< 0.001
Calculation time (s)	43.9 ± 7.3^a^	41.9 ± 7.3^b^	35.7 ± 3.5^c^	30.7 ± 3.1^d^	28.7 ± 2.8^e^	< 0.001
Delivery time (s)	305.3 ± 15.5^a^	158.1 ± 0.5^b^	158.1 ± 0.5^b^	158.1 ± 0.5^b^	158.1 ± 0.5^b^	< 0.001
Beam-on time (s)	236.5 ± 15.8^a^	149.0 ± 0.0^b^	149.7 ± 2.6^b^	149.0 ± 0.0^b^	149.0 ± 0.0^b^	< 0.001
Overall workflow time*	472.0 ± 29.4^a^	936.9 ± 73.7^bc^	936.8 ± 76.7^b^	905.5 ± 73.7^bc^	892.7 ± 80.4^c^	< 0.001
Total MU	1733.3 ± 136.9^a^	816.9 ± 65.5^b^	816.6 ± 76.5^b^	823.0 ± 70.5^b^	806.1 ± 69.2^b^	< 0.001
Average MU^#^	192.6 ± 15.2^a^	408.4 ± 32.7^b^	408.3 ± 38.2^b^	411.5 ± 35.2^b^	403.1 ± 34.6^b^	< 0.001

Values are presented as mean ± standard deviation. *P*-values were calculated using Repeated Measures ANOVA or Friedman test. Superscripts (^a,b,c,d,e^): Different letters within the same row indicate significant differences (*P<* 0.005, Bonferroni corrected) between groups.

*Overall workflow time = optimization time + calculation time + delivery time.

^#^ Average MU: Mean MU per field/arc.

Significant differences were observed across all evaluated metrics (*all P< 0.001*). Regarding the optimization time, IMRT required a significantly shorter optimization time (122.8 ± 14.6 s) compared to all VMAT groups (all *P< 0.005*). Among the VMAT groups, the optimization time for VMAT_5_ (705.9 ± 79.1 s) was significantly shorter than that for VMAT_3_ (742.9 ± 74.8 s). For dose calculation time, a progressive and significant reduction was observed from VMAT_2_ through VMAT_5_ (41.9 ± 7.3 s to 28.7 ± 2.8 s, all *P< 0.005*), as indicated by the distinct superscript letters (a-e) in **Table 4**.

In terms of delivery efficiency, all VMAT groups demonstrated a significant reduction in both total delivery time and beam-on time compared to the IMRT group (*P< 0.005*). Specifically, the total delivery time was reduced from 305.3 ± 15.5 s in IMRT to approximately 158.1 s in VMAT groups, while the beam-on time decreased from 236.5 ± 15.8 s to approximately 149.0 s. No statistically significant differences were found among the VMAT groups for these two delivery time metrics (*P > 0.005*).

When optimization time, dose-calculation time, and delivery time were combined, the overall workflow time was significantly shorter for IMRT than for all VMAT strategies (472.0 ± 29.4 s vs. 892.7–936.9 s, *P* < 0.001). Among the VMAT groups, VMAT_5_ had a significantly shorter overall workflow time than VMAT_3_, whereas no significant differences were observed for the remaining pairwise comparisons after Bonferroni correction. Thus, although VMAT substantially reduced treatment delivery time, this advantage did not compensate for its longer optimization time.

Regarding the monitor units (MU), the total MU for the IMRT group (1733.3 ± 136.9) was significantly higher than those of the VMAT groups (ranging from 806.1 to 823.0, *P< 0.005*).

### 3.4 Verification of plan deliverability

**Figure 5:**
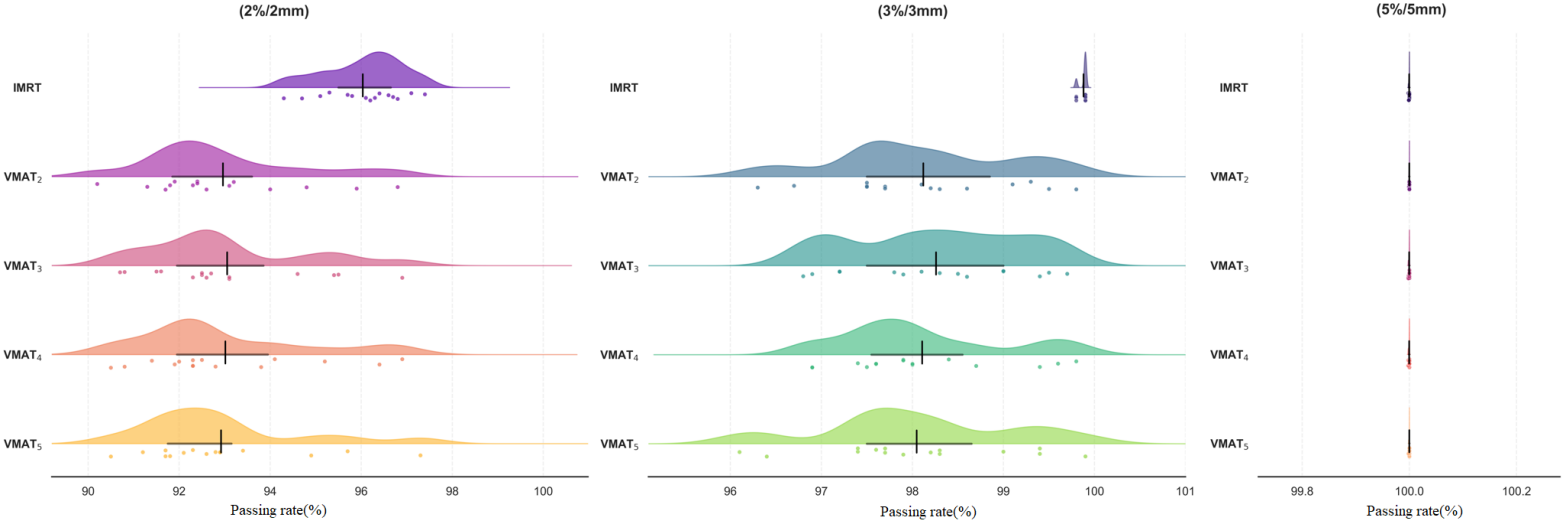
Distribution of Gamma passing rates for IMRT and VMAT plans under 2%/2mm,3%/3mm and 5%/5mm criteria.

The gamma passing rates under the 2%/2 mm, 3%/3 mm, and 5%/5 mm criteria are summarized in **Table 5** and **Figure 5**. Under the most stringent 2%/2 mm criterion, IMRT achieved a significantly higher passing rate than all VMAT groups (96.03 ± 0.88% vs. 92.92%–93.06%, P< 0.001). No statistically significant differences were observed among the VMAT_2_, VMAT_3_, VMAT_4_, and VMAT_5_ groups.

**Table 5:** Results of secondary dose verification and Gamma passing rates for IMRT and VMAT plans.

Criteria	IMRT	VMAT_2_	VMAT_3_	VMAT_4_	VMAT_5_	*P*-value
5%/5mm	100.00 ± 0.00^a^	100.00 ± 0.00^a^	100.00 ± 0.00^a^	100.00 ± 0.00^a^	100.00 ± 0.00^a^	1
3%/3mm	99.88 ± 0.04^a^	98.12 ± 0.82^b^	98.26 ± 0.81^b^	98.11 ± 0.77^b^	98.05 ± 0.86^b^	< 0.001
2%/2mm	96.03 ± 0.88^a^	92.96 ± 1.76^b^	93.056 ± 1.80^b^	93.01 ± 1.91^b^	92.92 ± 1.80^b^	< 0.001

Data are presented as mean ± standard deviation. *P*-values were determined by the Friedman test with Bonferroni-corrected *post-hoc* comparisons. Within each row, means labeled with different superscript letters (^a,b^) are significantly different (*P< 0.005*).

Similarly, under the 3%/3 mm criterion, the passing rate for IMRT was significantly higher than those for all VMAT groups (99.88 ± 0.04% vs. 98.05%–98.26%, P< 0.001), whereas the four VMAT groups remained comparable. Under the permissive 5%/5 mm criterion, all plans achieved a passing rate of 100%, confirming the absence of gross discrepancies but providing no meaningful discrimination among the planning strategies.

## 4 Discussion

The present study retrospectively compared the dosimetric quality, plan generation efficiency, delivery time, and deliverability of IMRT with VMAT plans optimized at angular resolutions of 2°, 3°, 4°, and 5° in the context of Ethos-based oART for cervical cancer. All strategies produced clinically acceptable plans meeting PTV coverage goals and OAR constraints. However, IMRT demonstrated superior target dose homogeneity (HI 0.07 ± 0.01 vs. 0.09 - 0.10 in VMAT), better high-dose sparing of critical OARs (including rectum V_40_, bladder D_mean_/V_40_/D_2cc_, and marrow V_30_), and substantially shorter plan optimization and calculation times. In contrast, VMAT provided clear advantages in beam-on time and total delivery time, with no significant differences in deliverability (gamma passing rates) across angular resolutions.

The contribution of the present study should be interpreted as comparative and platform-specific rather than methodological. The investigated beam arrangements and optimization options are components of the commercial Ethos planning environment, and the study does not propose a new optimization algorithm or delivery technique. Its contribution lies in the controlled evaluation of these available options under identical patient-specific anatomy and clinical goals, together with an integrated analysis of dosimetry, plan-generation time, treatment-delivery time, overall workflow time, and secondary verification. Importantly, the results demonstrate that the conventional delivery-time advantage of VMAT should not be considered in isolation in the online adaptive setting. Although VMAT approximately halved the treatment delivery time, its substantially longer optimization time resulted in a longer combined optimization, calculation, and delivery interval than IMRT. This workflow-level trade-off, together with the limited dosimetric effect of changing VMAT angular resolution from 2° to 5°, represents the principal new information provided by the present study.

The integration of Ethos-based oART for cervical cancer places a critical emphasis on workflow efficiency, as intra-fractional motion - driven by physiological changes such as bladder filling and rectal distension - can cause target displacement and compromise dose delivery if the interval from imaging to beam completion is prolonged (6, 12). Although VMAT has traditionally been preferred over IMRT in conventional pelvic radiotherapy for its faster delivery and often superior conformity (13, 14), our findings on the Ethos platform indicate a context-specific preference for fixed-field IMRT in oART workflows. This preference arises primarily from the IOE of the platform, which appears to handle static gantry fluence optimization more rapidly and effectively than the complex coupled variables of gantry speed and MLC dynamics required for VMAT.

In non-adaptive settings, multiple studies have reported advantages for VMAT over IMRT in cervical cancer, including reductions in rectum V_40_ and shorter delivery times (15). A meta-analysis by Bai W et al. found VMAT significantly protective for the rectum compared to IMRT, with no major differences in bladder or bowel doses, alongside substantial reductions in beam-on time and monitor units (15). Similarly, other dosimetric comparisons in postoperative or definitive cervical cases have shown VMAT achieving comparable or improved target conformity and OAR sparing, particularly for rectum and bladder (16).

Previous Ethos studies have demonstrated the clinical feasibility of CBCT-guided online adaptive radiotherapy in pelvic treatment sites and have reported early experience regarding contouring accuracy, plan quality, and treatment time in prostate cancer (17, 18). The present study extends this experience by directly comparing the manufacturer-defined nine-field IMRT configuration with VMAT plans using different angular resolutions within the same Ethos IOE for cervical cancer.

However, our findings on the Ethos platform diverged from the conventional expectation that VMAT would provide equal or superior dosimetric quality. The default nine-field IMRT configuration achieved better target homogeneity and lower values for several high-dose and intermediate-dose OAR parameters, including rectum V_40_, bladder D_mean_, V_40_, and D_2cc_, bowel D_2cc_, and marrow V_30_. Several interacting platform-specific mechanisms may explain this finding. Although VMAT theoretically provides continuous angular modulation and a larger number of control points, these advantages do not automatically result in a superior dose distribution within a specific automated optimization environment. Under an identical prioritized clinical goals template, IMRT and VMAT have different effective optimization spaces. In IMRT, the IOE can optimize the fluence of each stationary beam direction independently. In VMAT, however, the apertures of neighboring control points are coupled by the requirements for continuous gantry rotation, deliverable MLC trajectories, and transitions between successive control points. These delivery constraints may reduce the extent to which the theoretical angular freedom of VMAT can be exploited by the current IOE.

Beam-angle distribution may also have contributed to the observed differences. The nine-field IMRT arrangement concentrates fluence modulation into a limited number of discrete directions, potentially facilitating high-dose avoidance of the rectum and bladder immediately adjacent to the PTV and improving dose uniformity within the relatively large central pelvic target. In contrast, dual full-arc VMAT distributes entrance and exit fluence across a broader range of gantry angles. This broader distribution may reduce selected intermediate-dose endpoints, consistent with the lower bowel V_30_ observed with VMAT, while providing less favorable high-dose sparing for structures closely abutting the target, consistent with the higher bowel D_2cc_ and rectal and bladder doses. Importantly, the modern O-ring architecture and dual-layer MLC support efficient delivery but do not independently ensure superior dosimetry, which remains dependent on the interaction among the clinical-goal hierarchy, optimization algorithm, beam geometry, control-point sampling, and machine-deliverability constraints. Because the internal behavior of the proprietary IOE and detailed modulation-complexity parameters were not directly evaluated, these explanations should be regarded as technically plausible hypotheses rather than demonstrated causal mechanisms.

Although the absolute dosimetric differences were modest, they may become relevant when accumulated over a multi-fraction treatment course. In particular, the lower marrow V_30_ observed with IMRT may be clinically meaningful for patients receiving concurrent chemotherapy, because pelvic bone marrow dose has been associated with an increased risk of acute hematologic toxicity (19, 20).

This study provides a detailed evaluation of angular resolution (2°-5°) in Ethos VMAT for adaptive use. Among the VMAT plans, finer resolutions (particularly VMAT_2_) yielded modest improvements in selected high-dose OAR metrics, including lower bowel D_2cc_ and rectum V_40_ compared with VMAT_5_, consistent with the theoretical expectation that denser control points allow more precise fluence approximation and dose fall-off refinement. Prior studies on VMAT gantry increments in cervical cancer support this trend, reporting that smaller increments (e.g., 10°-20°) generally enhance plan quality over coarser ones (e.g., 30°-40°), though the improvements diminish beyond a certain threshold, often with trade-offs in monitor units and delivery time (21, 22).

However, the dosimetric penalty from coarser resolutions (4°-5°) was limited and not always clinically dominant, with no significant differences in most PTV metrics or overall gamma passing rates. Meanwhile, coarser resolutions reduced optimization and calculation times (VMAT_5_, approximately 706 s optimization time and 29 s calculation time vs. higher for VMAT_2_), reflecting fewer control points and simpler interpolation. The approximately 30s optimization time and 13 s calculation time saving with VMAT_5_ is small relative to the overall workflow (approximately 15–35 min typical for oART sessions (23)), suggesting that if VMAT is pursued, resolutions of 2°-3° may better preserve OAR sparing without meaningfully compromising efficiency. Coarser settings (4°-5°) risk a “false economy,” where minor time gains come at the expense of accumulated high-dose exposure to rectum or bowel.

It should also be acknowledged that the planning configurations evaluated in this study do not encompass all strategies that may be used for treatment on the Ethos platform. In addition to the manufacturer-defined Ethos configurations, individualized IMRT beam arrangements with different numbers of fields, customized gantry and collimator angles, and alternative or additional VMAT arc configurations can be generated in Eclipse before treatment. Such customized strategies may improve plan quality for selected patient anatomies or alter the balance between plan-generation and delivery efficiency. However, these approaches were outside the scope of the present study, which intentionally standardized beam geometry and optimization settings to isolate the effects of delivery technique and VMAT angular resolution. Therefore, the present findings should not be extrapolated to all customized Eclipse-based planning strategies.

VMAT delivered clear advantages in beam-on time (approximately 149 s vs. 236 s for IMRT) and total delivery time (approximately 158 s vs. 305 s for IMRT), reducing the treatment-execution period and potentially limiting intra-fractional motion. From a practical clinical-workflow perspective, the shorter optimization time of IMRT may reduce the interval between daily imaging and treatment initiation, thereby limiting anatomical divergence associated with ongoing bladder filling, rectal variation, and cervix–uterus displacement (14, 24–26). Conversely, the shorter delivery time of VMAT may reduce anatomical changes occurring during treatment execution. Therefore, planning-strategy selection should consider the dominant workflow bottleneck rather than delivery time alone. However, because no patient-specific intra-fractional imaging was analyzed, these implications should be regarded as potential clinical considerations rather than effects directly demonstrated by the present study. Nevertheless, the study-defined overall workflow time included only optimization, dose calculation, and delivery and should not be equated with the complete clinical oART session time, which also includes imaging, contour review and editing, plan evaluation, and approval.

Gamma analysis showed that the ability to distinguish among planning strategies depended strongly on the evaluation criterion. As expected, the permissive 5%/5 mm criterion yielded passing rates of 100% for all plans and therefore provided little discriminatory value beyond confirming the absence of gross discrepancies. In contrast, the more stringent 2%/2 mm criterion produced greater separation between IMRT and VMAT, with IMRT achieving a significantly higher passing rate than all VMAT groups. A similar but less pronounced pattern was observed under the conventional 3%/3 mm criterion. No significant differences were identified among the four VMAT angular resolutions under either the 2%/2 mm or 3%/3 mm criterion, suggesting that the observed difference was primarily related to the delivery technique rather than the selected VMAT angular resolution.

This study has several limitations. Firstly, as a retrospective dosimetric planning study using simulation CT data, it did not involve actual online adaptive delivery. consequently, the comparisons do not account for real-world factors such as intra-fractional motion during the adaptive workflow, residual setup uncertainties, or interplay effects in VMAT delivery. Secondly, the analysis was purely dosimetric, without incorporating normal tissue complication probability (NTCP) or tumor control probability (TCP) modeling or any correlation with clinical toxicity or tumor control outcomes, limiting assessment of the practical significance of the observed differences in OAR sparing and homogeneity. Thirdly, this was a single-institution study including only 15 patients. Although each patient underwent all five planning strategies and therefore served as their own control, the resulting 75 plans represented paired repeated measurements rather than independent observations. The repeated-measures design reduced variability associated with inter-patient anatomical differences but could not fully compensate for the limited number of independent patients. Consequently, the small cohort may have limited the ability to detect subtle differences among the VMAT angular resolutions and restricted the generalizability of the findings. Larger, preferably multicenter studies are required to validate these platform-specific results.

Efficiency metrics are specific to the current software version; future updates (such as GPU acceleration) could reduce VMAT optimization times and shift the workflow balance. Future research should prioritize prospective clinical studies that implement these planning strategies in real-time Ethos oART workflows, with endpoint assessments including acute and late toxicity rates, patient-reported outcomes, and accumulated delivered dose via dose accumulation techniques. Multi-center validation with larger cohorts would help confirm the platform-specific findings and explore hybrid IMRT-VMAT approaches or adaptive resolution strategies tailored to individual patient anatomy and motion patterns.

## 5 Conclusion

In this retrospective planning study, the manufacturer-defined 9-field IMRT configuration achieved better target homogeneity and improved sparing of several OARs, whereas VMAT provided substantially shorter treatment delivery time. However, when optimization, dose calculation, and delivery times were combined, IMRT showed a shorter overall workflow time than all VMAT strategies. Differences among VMAT angular resolutions of 2°–5° were generally modest, and coarser angular did not overcome the longer VMAT optimization time. These findings should be interpreted as a platform- and software-version-specific benchmark rather than evidence of the general superiority of one technique.

## Data Availability

The raw data supporting the conclusions of this article will be made available by the authors, without undue reservation.

## References

[1] Sung H Ferlay J Siegel RL Laversanne M Soerjomataram I Jemal A . Global cancer statistics 2020: GLOBOCAN estimates of incidence and mortality worldwide for 36 cancers in 185 countries. CA: A Cancer J For Clin. (2021) 71:209–49. doi: 10.3322/caac.21660 33538338

[2] Supriya C Nilesh R Mayuri C Sadhana K Reena E Tapas D . Late toxicity within a phase III clinical trial of IG-IMRT in cervix cancer (PARCER): Reanalysis with time weighted adverse event reporting (MOSES). Radiotherapy Oncol. (2022) 177:16–20. doi: 10.1016/j.radonc.2022.10.013 36270474

[3] Dröge LH von Sivers FF Schirmer MA Wolff HA . Conventional 3D conformal radiotherapy and volumetric modulated arc therapy for cervical cancer: Comparison of clinical results with special consideration of the influence of patient-and treatment-related parameters. Strahlenther Onkol. (2021) 197:520–7. doi: 10.1007/s00066-021-01782-5 PMC8154751 33938967

[4] Hasselle MD Rose B . Clinical outcomes of intensity-modulated pelvic radiation therapy for carcinoma of the cervix. Int J Radiat Oncol Biol Phys. (2011) 80:1436–45. doi: 10.1016/j.ijrobp.2010.04.041 20708346

[5] Corradini S Alongi F Andratschke N Belka C Boldrini L Cellini F . MR-guidance in clinical reality: Current treatment challenges and future perspectives. Radiat Oncol. (2019) 14:92. doi: 10.1186/s13014-019-1308-y 31167658 PMC6551911

[6] Zeng Z Zhu J Wang Z Wang G Yan J Zhang F . Pelvic target volume inter-fractional motion during radiotherapy for cervical cancer with daily iterative cone beam computed tomography. Radiat Oncol. (2024) 19:48. doi: 10.1186/s13014-024-02438-1 38622628 PMC11017626

[7] Sibolt P Andersson LM Calmels L Sjöström D Bjelkengren U Geertsen P . Clinical implementation of artificial intelligence-driven cone-beam computed tomography-guided online adaptive radiotherapy in the pelvic region. Phys Imaging Radiat Oncol. (2021) 17:1–7. doi: 10.1016/j.phro.2020.12.004 33898770 PMC8057957

[8] Byrne M Archibald‐Heeren B Hu Y Teh A Beserminji R Cai E . Varian ethos online adaptive radiotherapy for prostate cancer: Early results of contouring accuracy, treatment plan quality, and treatment time. J Appl Clin Med Phys. (2022) 23:e13479. doi: 10.1002/acm2.13479 34846098 PMC8803282

[9] Berger T Godart J Jagt T Vittrup AS Fokdal LU Lindegaard JC . Dosimetric impact of intrafraction motion in online-adaptive intensity modulated proton therapy for cervical cancer. Int J Radiat Oncol Biol Phys. (2021) 109:1580–7. doi: 10.1016/j.ijrobp.2020.11.037 33227442

[10] Small Jr W Mell LK Anderson P Creutzberg C De Los Santos J Gaffney D . Consensus guidelines for delineation of clinical target volume for intensity-modulated pelvic radiotherapy in postoperative treatment of endometrial and cervical cancer. Int J Radiat Oncol Biol Phys. (2008) 71:428–34. doi: 10.1016/j.ijrobp.2007.09.042 18037584 PMC2752724

[11] Gay HA Barthold HJ O’Meara E Bosch WR El Naqa I Al-Lozi R . Pelvic normal tissue contouring guidelines for radiation therapy: A Radiation Therapy Oncology Group consensus panel atlas. Int J Radiat Oncol Biol Phys. (2012) 83:e353–62. doi: 10.1016/j.ijrobp.2012.01.023 22483697 PMC3904368

[12] Wang GY Chen YN Sun YL . Impact evaluation of intra-fractional variation on online adaptive radiotherapy for postoperative cervical and endometrial cancer. World J Clin Oncol. (2025) 16:111601. doi: 10.5306/wjco.v16.i12.111601 41480167 PMC12754121

[13] Cozzi L Dinshaw KA Shrivastava SK . A treatment planning study comparing volumetric arc modulation with RapidArc and fixed field IMRT for cervix uteri radiotherapy. Radiotherapy Oncol. (2008) 89:180–91. doi: 10.1016/j.radonc.2008.06.013 18692929

[14] Wang Y Wang M Zhu L . The impact of bladder volume on dosimetric outcomes in VMAT for cervical cancer patients after surgery. J Gynecologic Oncol. (2025) 36:e65. doi: 10.3802/jgo.2025.36.e65 40223551 PMC12226321

[15] Bai W Kou C Yu W Li Y Hua W Yu L . Dosimetric comparison of volumetric-modulated arc therapy and intensity-modulated radiation therapy in patients with cervical cancer: A meta-analysis. OncoTargets Ther. (2018) 11:7179–86. doi: 10.2147/ott.s178336 30425510 PMC6203086

[16] Huang J Gu F Ji T Zhao J Li G . Pelvic bone marrow sparing intensity modulated radiotherapy reduces the incidence of the hematologic toxicity of patients with cervical cancer receiving concurrent chemoradiotherapy: A single-center prospective randomized controlled trial. Radiat Oncol. (2020) 15:180. doi: 10.1186/s13014-020-01606-3 32727497 PMC7389381

[17] Mell LK Schomas DA Salama JK Devisetty K Aydogan B Miller RC . Association between bone marrow dosimetric parameters and acute hematologic toxicity in anal cancer patients treated with concurrent chemotherapy and intensity-modulated radiotherapy. Int J Radiat Oncol Biol Phys. (2008) 70:1431–7. doi: 10.1016/j.ijrobp.2007.08.074 17996390

[18] Rose BS Aydogan B Liang Y Yeginer M Hasselle MD Dandekar V . Normal tissue complication probability modeling of acute hematologic toxicity in cervical cancer patients treated with chemoradiotherapy. Int J Radiat Oncol Biol Phys. (2011) 79:800–7. doi: 10.1016/j.ijrobp.2009.07.839 20400238 PMC2907446

[19] Chen A Li Z Chen L Lin M Li B Chen F . The influence of increment of gantry on VMAT plan quality for cervical cancer. J Radiat Res Appl Sci. (2019) 12:447–54. doi: 10.1080/16878507.2019.1707400 37339054

[20] Natraj M Pawaskar PN Chairmadurai A . Dosimetric characteristics of VMAT plans with respect to a different increment of gantry angle size for Ca cervix. J Radiother Pract. (2022) 21:92–6. doi: 10.1017/s146039692000093x 41292463

[21] Shen C Visak J Godley A Lin MH . Overview of artificial-intelligence driven adaptive therapy workflow. In: Artificial Intelligence in Adaptive Radiation Therapy. IOP Publishing, Bristol, UK (2025). p. 7-1–7-19.

[22] Byun DJ Oh C Kim J Barbee D Long M Fuligni G . Bladder filling dynamics during online adaptive prostate stereotactic body radiotherapy: Rationale for using an empty bladder workflow for treatment. Radiotherapy Oncol. (2025) 209:110978. doi: 10.1016/j.radonc.2025.110978 40466739

[23] Ahmad R Hoogeman MS Bondar M Dhawtal V Quint S De Pree I . Increasing treatment accuracy for cervical cancer patients using correlations between bladder-filling change and cervix–uterus displacements: Proof of principle. Radiotherapy Oncol. (2011) 98:340–6. doi: 10.1016/j.radonc.2010.11.010 21295877

[24] Bondar ML Hoogeman MS Mens JW Quint S Ahmad R Dhawtal G . Individualized nonadaptive and online-adaptive intensity-modulated radiotherapy treatment strategies for cervical cancer patients based on pretreatment acquired variable bladder filling computed tomography scans. Int J Radiat Oncol Biol Phys. (2012) 83:1617–23. doi: 10.1016/j.ijrobp.2011.10.011 22270164

[25] Paddick I . A simple scoring ratio to index the conformity of radiosurgical treatment plans. J Neurosurg. (2000) 93:219–22. doi: 10.3171/jns.2000.93.supplement_3.0219 11143252

[26] Gregoire V Mackie TR . Dose prescription, reporting and recording in intensity-modulated radiation therapy: A digest of the ICRU Report 83. Imaging Med. (2011) 3:367. doi: 10.2217/iim.11.22 21802333

