# Supplementary material for: Platform-specific comparison of IMRT and VMAT angular resolution for Ethos-based online adaptive radiotherapy in cervical cancer
Source: Front Oncol. 2026 Aug 5;16:1888330. doi: 10.3389/fonc.2026.1888330 (PMC13485773; doi:10.3389/fonc.2026.1888330)
Supplement: Supplementary file 1 [file Table1.docx]

Supplementary Table S1. Detailed optimization template and clinical dose constraints used for cervical cancer planning.

| Type | Structure | Metric | Clinical Constraint (Limit) | Optimization Priority |
| --- | --- | --- | --- | --- |
| Target | PTV | Coverage (V_50.4Gy_) | ≥ 95 % | 1-Most Important |
|  |  | D_max_ | < 110 % | 1-Most Important |
| Organs at Risk | Bladder | D_2cc_ | ≤53 Gy | 2-Very Important |
|  |  | D_mean_ | ≤40 Gy | 2-Very Important |
|  |  | V_40Gy_ | ≤ 55 % | 2-Very Important |
|  | Rectum | D_2cc_ | ≤53 Gy | 2-Very Important |
|  |  | D_mean_ | ≤40 Gy | 2-Very Important |
|  |  | V_40Gy_ | ≤ 55 % | 2-Very Important |
|  | Bowel | V_30Gy_ | ≤ 50 % | 2-Very Important |
|  |  | D_2cc_ | ≤53 Gy | 2-Very Important |
|  | Marrow | V_30Gy_ | ≤ 55 % | 2-Very Important |
|  |  | D_90%_ | ≤ 18 Gy | 2-Very Important |
|  | Femoral Heads | D_mean_ | ≤ 25 Gy | 3-Important |
|  |  | V_40Gy_ | ≤ 5 % | 3-Important |

Abbreviations: PTV = Planning Target Volume; D_max_ = Maximum Dose; D_mean_ = Mean Dose; V_xGy_ = Volume receiving at least x Gy; D_xcc_ = Minimum dose received by the most irradiated x cc volume.

*The same clinical goals template and priority order were applied to all five planning strategies. No manual adjustment of goal priorities or additional optimization objectives was performed during plan generation.
